# Supplementary material for: Selective plasticity of callosal neurons in the adult contralesional cortex following murine traumatic brain injury
Source: Nat Commun. 2022 May 12;13:2659. doi: 10.1038/s41467-022-29992-0 (PMC9098892; doi:10.1038/s41467-022-29992-0)
Supplement: Supplementary file 1 — Supplementary Information [file 41467_2022_29992_MOESM1_ESM.pdf]

Supplementary Information file

Selective plasticity of callosal neurons in the adult contralesional cortex following murine traumatic brain injury.

Laura Empl#, Alexandra Chovsepian#, Maryam Chahin, Wing Yin Vanessa Kan, Valérie Van Steenbergen, Julie Fourneau, Sanofer Weidinger, Maite Marcantoni, Alexander Ghanem, Peter Bradley, Karl Klaus Conzelmann, Ruiyao Cai, Alireza Ghasemigharagoz, Ali Ertürk, Ingrid Wagner, Mario Kreutzfeldt, Doron Merkler, Sabine Liebscher and Florence M Bareyre

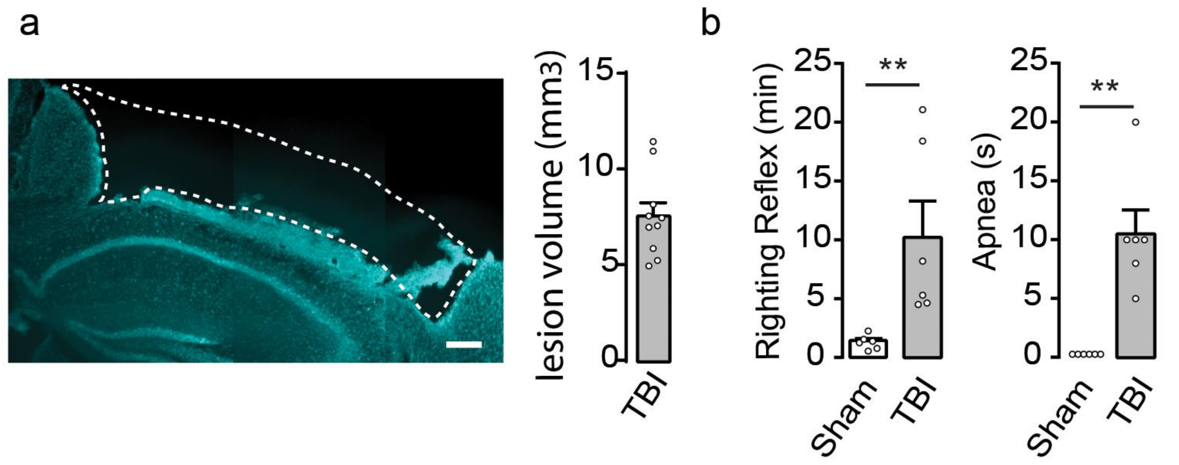

Supplementary Figure 1. Lesion volume, righting reflex and apnea and following moderate traumatic brain injury. (**a**) Representative confocal image of the lesion site 42 days following a moderate traumatic brain injury and quantification (mean ±SEM) of lesion volume in mm^3 (n=10 animals). Scale bar equals 100µm. (**b**) Quantification (mean ±SEM) of righting reflex and apnea following moderate traumatic brain injury. p=0.0022 for the righting reflex; p=0.0022 for the apnea. Mann Whitney 2-tailed test. n= 6 independent animals per group. Source data are provided as a Source Data file.

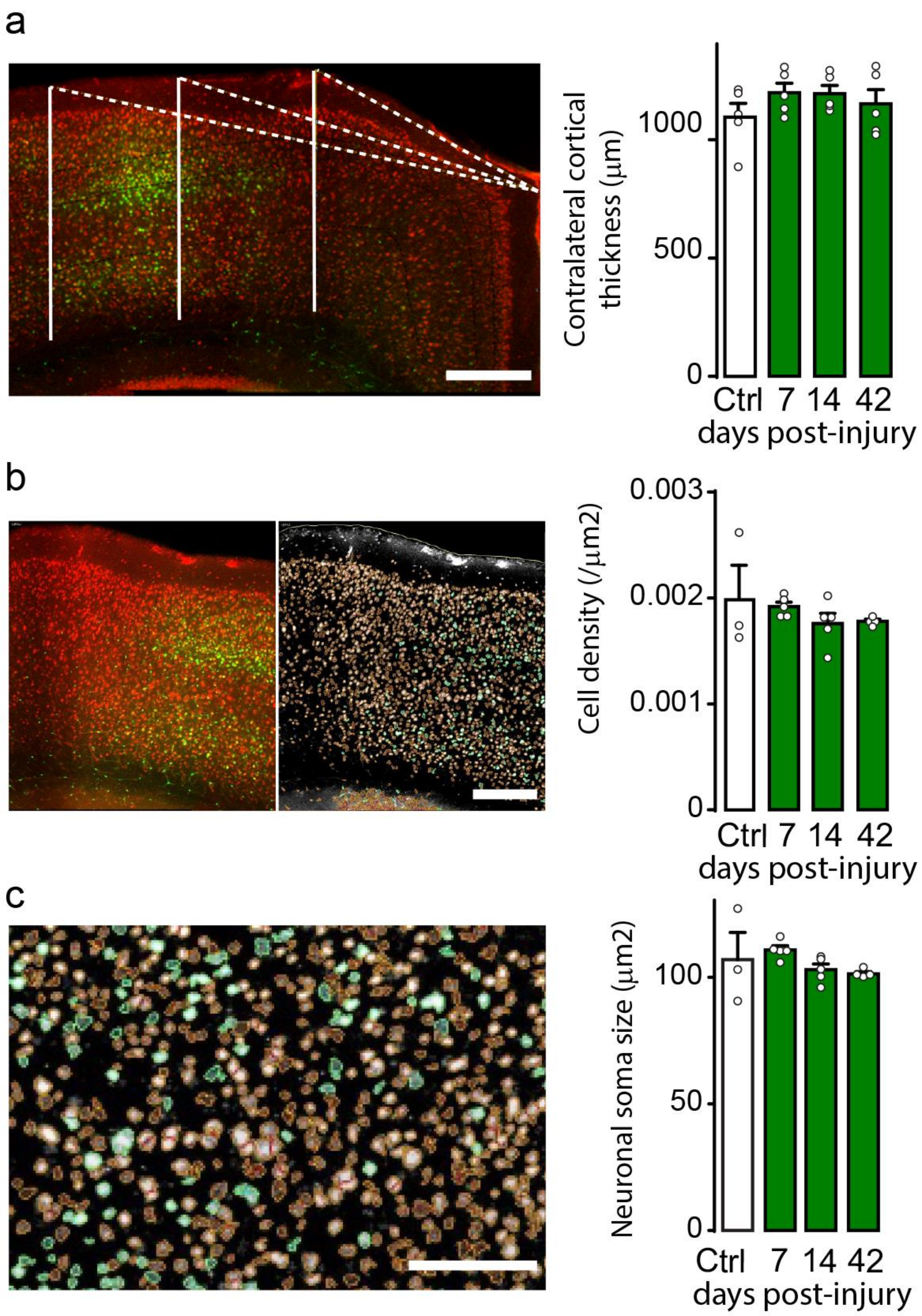

Supplementary Figure 2. Moderate traumatic brain injury does not induce changes in contralesional cortical thickness, contralesional neuronal density or contralesional neuronal soma size. (**a**) Representative confocal image of the contralesional cortical area to the brain injury. White lines indicate the distance between the end of layer VI and the beginning of layer I at three standard different locations in the mediolateral direction (measured as indicated with dashed lines; green: retrogradely labeled callosal neurons; red: neurotrace).

Scale bar: 350μm. Quantification (mean ±SEM) of the cortical thickness in control (white column) and injured animals (green column) at different time points following TBI. (**b**) Representative confocal image of the cortex (left) and the identification of neurons (right). Scale bar equals 300μm. Quantification (mean ±SEM) of cortical neuronal density in control (white column) and injured (green column) animals at different time points following TBI. (**c**) Representative confocal image of cortical neurons to calculate some size. Scale bar equals 450μm. Quantification (mean ±SEM) of neuronal soma size in control (white column) and injured (green column) animals at different time points following TBI. n=3-5 independent animals for all evaluations. Source data are provided as a Source Data file.

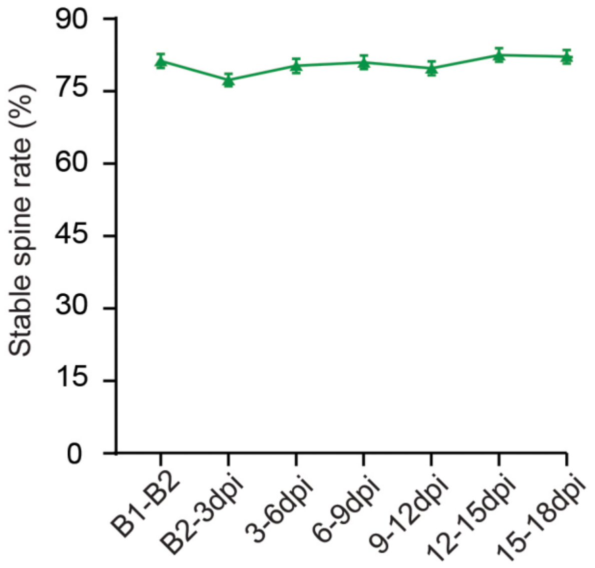

Supplementary Figure 3. The rate of stable spines in contralesional callosal neurons does not vary overtime following brain injury. Mean percentage of the rate of stable spines on dendrites of layer V neurons in GFP-M mice. Data come from 81 dendritic stretches, 10191 spines followed over time and n=4 animals. Data are analyzed with two-way repeated measure (RM) ANOVA followed by Bonferroni test. Source data are provided as a Source Data file.
