# Supplementary material for: Selective plasticity of callosal neurons in the adult contralesional cortex following murine traumatic brain injury
Source: Nat Commun. 2022 May 12;13:2659. doi: 10.1038/s41467-022-29992-0 (PMC9098892; doi:10.1038/s41467-022-29992-0)
Supplement: Supplementary file 2 — Reporting Summary [file 41467_2022_29992_MOESM2_ESM.pdf]

nature research

Corresponding author(s): F. Bareyre (florence.bareyre@med.uni-muenchen.de)

Last updated by author(s): March 16, 2022

Reporting Summary

Nature Research wishes to improve the reproducibility of the work that we publish. This form provides structure for consistency and transparency in reporting. For further information on Nature Research policies, see our Editorial Policies and the Editorial Policy Checklist.

Statistics

For all statistical analyses, confirm that the following items are present in the figure legend, table legend, main text, or Methods section.

n/a	Confirmed	
☐	☒	The exact sample size (n) for each experimental group/condition, given as a discrete number and unit of measurement
☐	☒	A statement on whether measurements were taken from distinct samples or whether the same sample was measured repeatedly
☐	☒	The statistical test(s) used AND whether they are one- or two-sided<br>*Only common tests should be described solely by name; describe more complex techniques in the Methods section.*
☒	☐	A description of all covariates tested
☒	☐	A description of any assumptions or corrections, such as tests of normality and adjustment for multiple comparisons
☐	☒	A full description of the statistical parameters including central tendency (e.g. means) or other basic estimates (e.g. regression coefficient) AND variation (e.g. standard deviation) or associated estimates of uncertainty (e.g. confidence intervals)
☐	☒	For null hypothesis testing, the test statistic (e.g. F, t, r) with confidence intervals, effect sizes, degrees of freedom and P value noted<br>*Give P values as exact values whenever suitable.*
☒	☐	For Bayesian analysis, information on the choice of priors and Markov chain Monte Carlo settings
☒	☐	For hierarchical and complex designs, identification of the appropriate level for tests and full reporting of outcomes
☒	☐	Estimates of effect sizes (e.g. Cohen's d, Pearson's r), indicating how they were calculated

Our web collection on statistics for biologists contains articles on many of the points above.

Software and code

Policy information about availability of computer code

Data collection	FV1000 confocal microscope fluoview von Olympus;Leica DM4B upright fluorescence microscope;Leica TCS SP8 confocal microscope. Olympus FV1000MPE two photon microscope with Mai Tai DeepSee femtosecond plused Ti:Sapphire laser (Spectraphysics); Resonant scanning two-photon microscope (Hyperscope, Scientifica, equipped with an 8kHz resonant scanner) with Ti:Sapphire laser with a DeepSee pre-chirp unit (Spectra Physics MaiTai eHP). Light-sheet microscope Ultramicroscope II (LaVision BioTec).
Data analysis	Image J (1.52h), Definiens Developer XD (v2.7.0), Huygens Essential (16.05), Neuronstudio(version 0.9.92), Matlab(R2018b) with custom script (already published inLiebscher, S., Keller, G. B., Goltstein, P. M., Bonhoeffer, T. & Hübener, M. Selective Persistence of Sensorimotor Mismatch Signals in Visual Cortex of Behaving Alzheimer's Disease Mice. Curr. Biol. 26, 956–964 (2016).), Prism (7.01), MBF Stereo Ivestigator 2017.

For manuscripts utilizing custom algorithms or software that are central to the research but not yet described in published literature, software must be made available to editors and reviewers. We strongly encourage code deposition in a community repository (e.g. GitHub). See the Nature Research guidelines for submitting code & software for further information.

Data

Policy information about availability of data

All manuscripts must include a data availability statement. This statement should provide the following information, where applicable:

- Accession codes, unique identifiers, or web links for publicly available datasets
- A list of figures that have associated raw data
- A description of any restrictions on data availability

Source data are provided with this paper. All the XX data generated in this study have been deposited in the Zenodo database under accession code ZZ [add hyperlink here] including supplementary data.

Field-specific reporting

Please select the one below that is the best fit for your research. If you are not sure, read the appropriate sections before making your selection.

- [x] Life sciences
- [ ] Behavioural & social sciences
- [ ] Ecological, evolutionary & environmental sciences

For a reference copy of the document with all sections, see nature.com/documents/nr-reporting-summary-flat.pdf

Life sciences study design

All studies must disclose on these points even when the disclosure is negative.

Sample size	No statistical methods were used to pre-determine sample size but our sample sizes are similar to those reported in previous publications. Falkner, S. et al. Nature 539, 248–253 (2016).Bradley, P. M. et al. J. Exp. Med. 216, 2503–2514 (2019).Liebscher, S.et al. Curr. Biol. 26, 956–964 (2016).Ertürk A et al. J Neurosci. (2016).
Data exclusions	Pre-established exclusion criteria were used in this study. Mice were excluded when they could not be imaged at all time points due to poor cranial window quality for two photon in vivo imaging (Fig. 1 and 4; Suppl Fig. 2). For ex vivo imaging, mice or tissue showing insufficient lableling or staining or structures showing insufficient signal to noise ratios were not analyzed (Fig. 2; Suppl Fig. 1, 3&4). For connectivity data mice with an insufficient labeling of starter cells (or inadequate distribution per layer) were not included in the analysis (Fig. 3).
Replication	All experiments in this study include at least 4 biological replicates. The number of replicates (ns) is mentioned in the text or figure legend.
Randomization	Mice were randomly allocated to groups in all experiments.
Blinding	Data collection and analysis was performed blind to the conditions of the experiment, unless this was not possible (for exemple, longitudinal in vivo imaging data that were collected unblindly from time points but analyzed blindly).

Reporting for specific materials, systems and methods

We require information from authors about some types of materials, experimental systems and methods used in many studies. Here, indicate whether each material, system or method listed is relevant to your study. If you are not sure if a list item applies to your research, read the appropriate section before selecting a response.

Materials & experimental systems

n/a	Involved in the study	
[ ]	[x]	Antibodies
[x]	[ ]	Eukaryotic cell lines
[x]	[ ]	Palaeontology and archaeology
[ ]	[x]	Animals and other organisms
[x]	[ ]	Human research participants
[x]	[ ]	Clinical data
[x]	[ ]	Dual use research of concern

Methods

n/a	Involved in the study	
[x]	[ ]	ChIP-seq
[x]	[ ]	Flow cytometry
[x]	[ ]	MRI-based neuroimaging

Antibodies

Antibodies used

Anti NeuN mouse antibody- Thermo Fisher Scientific. MAB377MI
goat anti-mouse AlexaFluor® 594 antibody. Thermo Fisher. A-11020
NeuroTrace 435. Invitrogen/Thermo Fischer Scientific N21479
Fluorogold, Fluorochrome LLC
RFP-Booster Atto647N, Chromotek. rba647n-100
chicken anti-GFP Ab, Abcam ab13970
Goat anti chicken Alexa Fluor 488, Abcam ab150169
rabbit anti-RFP antibody (abcam, ab124754)
anti-rabbit HRP (Dako,K4003)
goat-anti-mouse IgG Fab-fragments (Jackson ImmunoResearch, 115-007-003)
mouse IgG2b anti-Gephyrin antibody (Santa Cruz, sc-25311)
mouse IgG1 anti-GAD65/67 antibody (Santa Cruz, sc-365180)
chicken anti-Homer1 antibody (Synaptic System, 1600006)
mouse IgG1 anti- BNPI antibody (Santa Cruz, sc-377425)
DAPI (Invitrogen, D1306)
Goat anti Mouse IgG2b Alexa Fluor 488 (Invitrogen Antibodies A21141)
Goat anti Mouse IgG1 Alexa Fluor 647 (Invitrogen Antibodies A21240)

Goat anti Chicken IgY Alexa Fluor 488 (Invitrogen Antibodies A11039)
Opal-570 (Akoya, FP1488001KT)

Validation

All antibodies used in this study are commercially available and therefore validated by the manufacturer. Here are the validation references:

Anti NeuN: https://www.fishersci.com/shop/products/anti-neun-antibody-clone-a60/mab377mi; https://www.ncbi.nlm.nih.gov/pmc/articles/PMC3009842/; https://www.ncbi.nlm.nih.gov/pmc/articles/PMC4657622/
goat anti mouse 594: https://www.thermofisher.com/antibody/product/Goat-anti-Mouse-IgG-H-L-Cross-Adsorbed-Secondary-Antibody-Polyclonal/A-11020. https://pubmed.ncbi.nlm.nih.gov/29908948/; https://pubmed.ncbi.nlm.nih.gov/17428831/
Neurotrace 435: https://www.thermofisher.com/order/catalog/product/N21479?de&en#/N21479?de&en; https://pubmed.ncbi.nlm.nih.gov/18256272/; https://pubmed.ncbi.nlm.nih.gov/30759385/
Fluorogold:https://fluorochrome.com/flouro-gold/; Tosolini A.P., Mohan R, Morris R., “Targeting the full length of the motor end plates regions in the mouse forelimb increases the uptake of Fluoro-Gold into corresponding spinal cord motor neurons,” Frontiers in Neurology (2013) 1-10.;Schmued, L.C., “Anti-retrograde and retrograde neuroanatomical tract tracing with fluorescent compounds,” Neuroscience Protocols, 94-050-02 (1994) 1-15.
RFP-BOOSTer ATTO647N: https://www.chromotek.com/products/detail/product-detail/rfp-booster/ ;https://www.ncbi.nlm.nih.gov/pmc/articles/PMC6494982/
chicken anti GFP: https://www.abcam.com/gfp-antibody-ab13970.html; https://pubmed.ncbi.nlm.nih.gov/31937758/; https://pubmed.ncbi.nlm.nih.gov/31967543/
Goat anti chicken alexa fluor 488:https://www.abcam.com/goat-chicken-igy-hl-alexa-fluor-488-ab150169.html; https://pubmed.ncbi.nlm.nih.gov/31797735/; https://pubmed.ncbi.nlm.nih.gov/32211098/
Jafari M, Schumacher AM, Snaidero N, Ullrich Gavilanes EM, Neziraj T, Kocsis-Jutka V, Engels D, Jürgens T, Wagner I, Weidinger JDF, Schmidt SS, Beltrán E, Hagan N, Woodworth L, Ofengeim D, Gans J, Wolf F, Kreutzfeldt M, Portugues R, Merkler D, Misgeld T, Kerschensteiner M (2021)
Phagocyte-mediated synapse removal in cortical neuroinflammation is promoted by local calcium accumulation.
Nat Neurosci. 2021 Jan 25. doi: 10.1038/s41593-020-00780-7. Epub ahead of print. PMID: 33495636.
rabbit anti-RFP (abcam, ab124754):Striebel JF et al. Microglia are not required for prion-induced retinal photoreceptor degeneration. Acta Neuropathol Commun 7:48 (2019). Liu X et al. Cell-Type-Specific Interleukin 1 Receptor 1 Signaling in the Brain Regulates Distinct Neuroimmune Activities. Immunity 50:317-333.e6 (2019).
anti-rabbit HRP (Dako,K4003): Sato N, Sakai N, Furukawa K, Takayashiki T, Kuboki S, Takano S, Ohira G, Matsubara H, Ohtsuka M. Yin Yang 1 regulates ITGAV and ITGB1, contributing to improved prognosis of colorectal cancer. Oncol Rep. 2022
Shinkawa T, Ohuchida K, Mochida Y, Sakihama K, Iwamoto C, Abe T, Ideno N, Mizuuchi Y, Shindo K, Ikenaga N, Moriyama T, Nakata K, Oda Y, Nakamura M. Subtypes in pancreatic ductal adenocarcinoma based on niche factor dependency show distinct drug treatment responses. J Exp Clin Cancer Res. 2022 Mar 10;41(1):89.
goat-anti-mouse IgG Fab-fragments (Jackson ImmunoResearch, 115-007-003):Shishido SN, Sayeed S, Courcoubetis G, Djaladat H, Miranda G, Pienta KJ, Nieva J, Hansel DE, Desai M, Gill IS, Kuhn P, Mason J. Characterization of Cellular and Acellular Analytes from Pre-Cystectomy Liquid Biopsies in Patients Newly Diagnosed with Primary Bladder Cancer. Cancers (Basel). 2022 Feb 1;14(3):758.
Weitzel AJ, Grunwald HA, Weber C, Levina R, Gantz VM, Hedrick SM, Bier E, Cooper KL. Meiotic Cas9 expression mediates gene conversion in the male and female mouse germline. PLoS Biol. 2021 Dec 23;19(12):e3001478.
mouse IgG2b anti-Gephyrin antibody (Santa Cruz, sc-25311):Manabe T, Rácz I, Schwartz S, Oberle L, Santarelli F, Emmrich JV, Neher JJ, Heneka MT. Systemic inflammation induced the delayed reduction of excitatory synapses in the CA3 during ageing. J Neurochem. 2021 Nov;159(3):525-542.
Wang J, Huang J, Li YQ, Yao S, Wu CH, Wang Y, Gao F, Xu MD, Huang GB, Zhao CQ, Wu JH, Zhang YL, Jiao R, Deng ZH, Jie W, Li HB, Xuan A, Sun XD. Neuregulin 1/ErbB4 signaling contributes to the anti-epileptic effects of the ketogenic diet. Cell Biosci. 2021 Feb 3;11(1):29.
mouse IgG1 anti-GAD65/67 antibody (Santa Cruz, sc-365180):Li Y, Su P, Chen Y, Nie J, Yuan TF, Wong AH, Liu F. The Eph receptor A4 plays a role in demyelination and depression-related behavior. J Clin Invest. 2022 Mar 10:e152187.
Lacaille H, Vacher CM, Penn AA. Preterm Birth Alters the Maturation of the GABAergic System in the Human Prefrontal Cortex. Front Mol Neurosci. 2022 Feb 3;14:827370.
chicken anti-Homer1 antibody (Synaptic System, 1600006): Inhibition of LRRK2 kinase activity promotes anterograde axonal transport and presynaptic targeting of α-synuclein.
Brzozowski CF, Hijaz BA, Singh V, Gcwensa NZ, Kelly K, Boyden ES, West AB, Sarkar D, Volpicelli-Daley LA
Acta neuropathologica communications (2021) 91: 180. 160 006 ICC, IHC; tested species: mouse
Pyk2 overexpression in postsynaptic neurons blocks amyloid β1-42-induced synaptotoxicity in microfluidic co-cultures.
Kilinc D, Vreulx AC, Mendes T, Flaig A, Marques-Coelho D, Verschoore M, Demiautte F, Amouyel P, , Eysert F, Dourlen P, et al.
Brain communications (2020) 22: fcaa139. 160 006 ICC; tested species: rat
mouse IgG1 anti- BNPI antibody (Santa Cruz, sc-377425): Branscome H, Khatkar P, Al Sharif S, Yin D, Jacob S, Cowen M, Kim Y, Erickson J, Brantner CA, El-Hage N, Liotta LA, Kashanchi F. Retroviral infection of human neurospheres and use of stem Cell EVs to repair cellular damage. Sci Rep. 2022 Feb 7;12(1):2019.
Contreras-García IJ, Gómez-Lira G, Phillips-Farfán BV, Pichardo-Macías LA, García-Cruz ME, Chávez-Pacheco JL, Mendoza-Torreblanca JG. Synaptic Vesicle Protein 2A Expression in Glutamatergic Terminals Is Associated with the Response to Levetiracetam Treatment. Brain Sci. 2021 Apr 23;11(5):531.
DAPI (Invitrogen, D1306): https://www.thermofisher.com/document-connect/document-connect.html?url=https%3A%2F%2Fassets.thermofisher.com%2FTFS-Assets%2FBID%2Fposters%2Fhuman-hepatoctye-3d-spheroids-hepatic-function-drug-toxicity-poster.pdf
Goat anti Mouse IgG2b Alexa Fluor 488 (Invitrogen Antibodies A21141): Radecki DZ, Samanta J. Isolation and culture of neural stem cells from adult mouse subventricular zone for genetic and pharmacological treatments with proliferation analysis. STAR Protoc. 2022 Feb 3;3(1):101153.
Kislev N, Mor-Yossef Moldovan L, Barak R, Egozi M, Benayahu D. MYH10 Governs Adipocyte Function and Adipogenesis through Its Interaction with GLUT4. Int J Mol Sci. 2022 Feb 21;23(4):2367.
Goat anti Mouse IgG1 Alexa Fluor 647 (Invitrogen Antibodies A21240): Goodwin K, Jaslove JM, Tao H, Zhu M, Hopyan S, Nelson CM.

Patterning the embryonic pulmonary mesenchyme. iScience. 2022 Jan 29;25(3):103838. Kurotsu S, Sadahiro T, Harada I, Ieda M. A biomimetic hydrogel culture system to facilitate cardiac reprogramming. STAR Protoc. 2022 Jan 22;3(1):101122.
Goat anti Chicken IgY Alexa Fluor 488 (Invitrogen Antibodies A11039):Liu FD, Duan HM, Hao F, Zhao W, Gao YD, Hao P, Yang ZY, Li XG. Biomimetic chitosan scaffolds with long-term controlled release of nerve growth factor repairs 20-mm-long sciatic nerve defects in rats. Neural Regen Res. 2022 May;17(5):1146-1155. Noguchi A, Huszár R, Morikawa S, Buzsáki G, Ikegaya Y. Inhibition allocates spikes during hippocampal ripples. Nat Commun. 2022 Mar 11;13(1):1280.
Opal-570 (Akoya, FP1488001KT): Radtke AJ, Kandov E, Lowekamp B, Speranza E, Chu CJ, Gola A, Thakur N, Shih R, Yao L, Yaniv ZR, Beuschel RT, Kabat J, Croteau J, Davis J, Hernandez JM, Germain RN. IBEX: A versatile multiplex optical imaging approach for deep phenotyping and spatial analysis of cells in complex tissues. Proc Natl Acad Sci U S A. 2020 Dec 29;117(52):33455-33465.

Animals and other organisms

Policy information about studies involving animals; ARRIVE guidelines recommended for reporting animal research

Laboratory animals	GFP-M mice (mixed gender randomized equally per group) and female C57Bl6J mice, eight to twelve weeks-old, were used for this study. All animals were housed under controlled standard housing conditions (dark/light cycle of 12h, temperature 22±2 degrees and humidity of 55±10%) and each animal experiment was carried out in accordance with German animal welfare guidelines and previously authorized by the local regulatory committees under the protocol number AZ:55.2.1.54-2532.135-15.
Wild animals	No wild animals were assessed in this paper.
Field-collected samples	No field-collected samples were assessed here.
Ethics oversight	All animal experiments were performed in accordance with the regulations of the local animal committee of the state of Bavaria (Regierung von Oberbayern) under the protocol number AZ:55.2.1.54-2532.135-15. .

Note that full information on the approval of the study protocol must also be provided in the manuscript.
