# Supplementary material for: Selective plasticity of callosal neurons in the adult contralesional cortex following murine traumatic brain injury
Source: Nat Commun. 2022 May 12;13:2659. doi: 10.1038/s41467-022-29992-0 (PMC9098892; doi:10.1038/s41467-022-29992-0)
Supplement: Supplementary file 4 — Description of Additional Supplementary Files [file 41467_2022_29992_MOESM4_ESM.pdf]

Title: Supplementary Movie 1:
Description: Movie of a cleared brain with trans-synaptic retrograde tracing using rabies virus to identify the general location of presynaptic inputs of callosal neurons in control animals (green label) and following traumatic brain injury (red label).
